# Tumorigenesis and peritoneal colonization from fallopian tube epithelium

**DOI:** 10.18632/oncotarget.3985

**Published:** 2015-05-04

**Authors:** Sharon L. Eddie, Suzanne M. Quartuccio, Eoghainin Ó hAinmhire, Georgette Moyle-Heyrman, Dan D. Lantvit, Jian-Jun Wei, Barbara C. Vanderhyden, Joanna E. Burdette

**Affiliations:** ^1^ Department of Medicinal Chemistry and Pharmacognosy, University of Illinois at Chicago, Chicago, IL, USA; ^2^ Department of Pathology, Feinberg School of Medicine, Northwestern University, Chicago, IL, USA; ^3^ Department of Cellular and Molecular Medicine, University of Ottawa, Ottawa, Ontario, Canada

**Keywords:** high-grade serous carcinoma, fallopian tube, PTEN, KRAS, p53, AKT

## Abstract

Ovarian cancer is the most lethal gynecological malignancy, primarily because its origin and initiation factors are unknown. A secretory murine oviductal epithelial (MOE) model was generated to address the hypothesis that the fallopian tube is an origin for high-grade serous cancer. MOE cells were stably altered to express mutation in p53, silence PTEN, activate AKT, and amplify KRAS alone and in combination, to define if this cell type gives rise to tumors and what genetic alterations are required to drive malignancy. Cell lines were characterized *in vitro* and allografted into mice. Silencing PTEN formed high-grade carcinoma with wide spread tumor explants including metastasis into the ovary. Addition of p53 mutation to PTEN silencing did not enhance this phenotype, whereas addition of KRAS mutation reduced survival. Interestingly, PTEN silencing and KRAS mutation originating from ovarian surface epithelium generated endometrioid carcinoma, suggesting that different cellular origins with identical genetic manipulations can give rise to distinct cancer histotypes. Defining the roles of specific signaling modifications in tumorigenesis from the fallopian tube/oviduct is essential for early detection and development of targeted therapeutics. Further, syngeneic MOE allografts provide an ideal model for pre-clinical testing in an *in vivo* environment with an intact immune system.

## INTRODUCTION

Ovarian cancer is a heterogeneous disease composed of several histotypes including mucinous, clear cell, endometrioid, low-grade and high-grade serous. In the United States, 23,000 women will be diagnosed and 14,000 will succumb to these diseases each year [1]. High-grade serous carcinoma (HGSC) is the most common and lethal histotype and is suggested to arise from the fallopian tube fimbria. Putative fallopian precursor, lesions are thought to arise from the mutation of p53, followed by subsequent oncogenic events that confer expansion into serous tubal intraepithelial carcinoma (STIC) and ultimately into HGSC [2]. HGSC are primarily diagnosed at the metastatic stage (75% of cases) and are treated with debulking surgery and a combination of paclitaxel and carboplatin, but patients often develop chemoresistance [3]. Defining the molecular events leading to HGSC carcinogenesis and progression from the fallopian tube is essential for identification of targets for early detection and personalized drug therapy.

Murine models of HGSC originating from the fallopian tube equivalent, the oviductal epithelium, reveal that these cells can give rise to tumors. These murine models utilize the PAX8-TetOn-Cre promoter to drive the deletion of PTEN, BRCA1 or 2, and mutation of p53 in combination, as well as deletion of PTEN together with deletion of p53 [4]. These models produced tumors that were highly metastatic, colonized the ovaries, and expressed oviductal markers. Interestingly, another murine model with floxed alleles of PTEN and KRAS developed high-grade endometrioid carcinoma when exposed to adenovirus encoding for Cre-recombinase regardless of the tissue type targeted (ovarian surface or oviductal epithelium [5]). A further model utilizing the MISRII promoter driving Cre-recombinase expression to delete PTEN alone or in combination with DICER in oviductal stromal cells forces a stromal-to-epithelial derived high-grade tumor type [6]. Unfortunately, despite the advantages of using the Cre-Lox system to generate murine models, the limited viral infectivity of the oviduct epithelium and the inability to target the oviduct specifically with promotor driven Cre-recombinase without impacting additional cell types could mask or confuse the question of cellular origin [7]. Furthermore, although p53 is mutated in 96-100% of HGSC tumors and is thought to be one of the earliest molecular changes in fallopian derived HGSC, animals with mutation in p53 alone have not yet been characterized. Additionally, mutation in p53 has not been examined with the loss of PTEN, in the absence of BRCA mutation, which is of interest as ∼70-80% of HGSC tumors are spontaneous and not the result of mutation or methylation in BRCA (11). Moreover, human fallopian cell models have demonstrated the importance of KRAS and c-myc in generating HGSC and mucinous histotypes from fallopian tube epithelium [8-10]. In these human models, p53 is either silenced or inactivated by SV40T, which hinders the ability to study the impact of gain-of-function p53 mutations, which represent the vast majority of mutations in HGSC.

The Cancer Genome Atlas Network demonstrated that several key pathways are altered in HGSC, yet many of them are not altered strictly through mutation [11]. For example, PTEN is rarely mutated in HGSC but instead tumors demonstrate a loss of heterozygosity. Similarly, tissue microarray studies demonstrate AKT activation through phosphorylation occurs early in tumorigenesis and is independent of PTEN, AKT, and PIK3Ca mutation [12, 13]. KRAS is also rarely mutated, but instead is amplified in many tumors. As many HGSC tumors are studied in a late stage, metastatic setting, it is unclear whether altered pathways are initiators of disease or if they are compounded genetic alterations.

The purpose of this study was to engineer clonogenic murine oviductal epithelial (MOE) models with individual and multiple pathway modifications to mimic changes detected in human HGSC tumors. The ability to study mutant p53 alone and in combination with several other events allows for clarification of its role and its ability to modify other pathways. Similarly, models were constructed with PTEN silenced, but not completely deleted, similar to loss of heterozygosity in HGSC and with AKT constitutively activated by myristolation. Lastly, the G12V mutation was utilized to activate KRAS function. These unique MOE cell models indicate that loss of PTEN alone is sufficient to drive tumorigenesis and widely spread metastasis in the peritoneal cavity. Mutation of p53 did not enhance this process, while mutation of KRAS significantly decreased survival time. These models indicate that specific mutation in oviductal epithelium can produce tumors with unique morphologies and specific secondary sites of tumor spread, including the ovary suggesting a role for this tissue in the dissemination of disease. Finally this study validates that novel MOE models can be utilized in syngeneic allografts allowing for preclinical therapeutic development in an immune-competent *in vivo* environment.

## RESULTS

### MOE cells stably expressing targets altered in HGSC patients

To examine the effects of genetic alterations similar to those observed in HGSC patients, MOE cells were stably transfected with mutation in p53 R273H (p53^R273H^), silenced PTEN via shRNA (PTEN^shRNA^), activation of AKT via myristolation (AKT^MYR^) and activating mutation in KRAS G12V (KRAS^G12V^) (Suppl. Table 1). In addition cell lines harboring multiple genetic modifications were created, as often multiple mutations are required for oncogenic transformation. These cells were validated to express targets of interest via western blotting (Suppl. Fig 1A) compared to parental wildtype and scrambled shRNA control MOE cell lines. Signaling downstream of genetic manipulations was also altered as expected, demonstrating that both the targets themselves and their signaling pathways were functionally impacted (Suppl. Fig 1B).

### Silencing PTEN increases MOE cell proliferation and addition of p53^R273H^, AKT^MYR^, or KRAS^G12V^ promotes *in vitro* transformation

**Figure 1:**
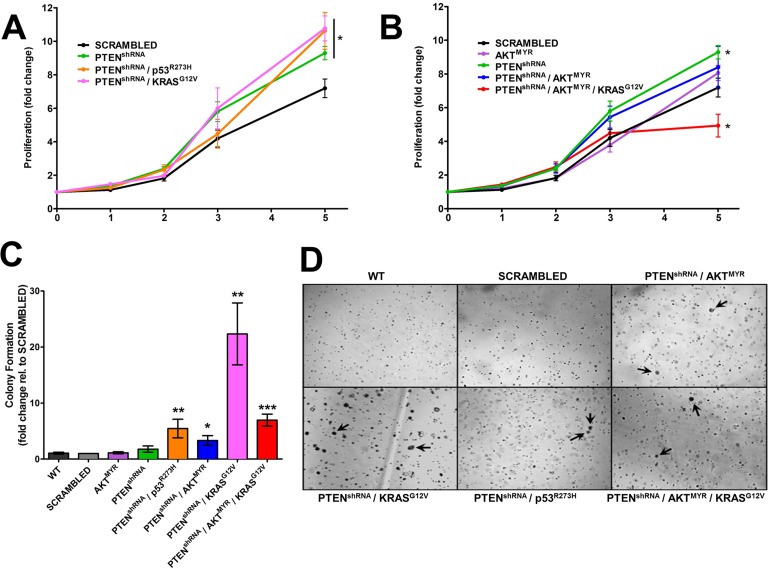
Genetically engineered MOE have tumorigenic characteristics *in vitro* **A.** MOE cells stably altered with PTEN^shRNA^, PTEN^shRNA^/p53^R273H^, and PTEN^shRNA^/KRAS^G12V^ were significantly more proliferative than scrambled shRNA transfected and parental MOE cells (data not shown) after 5 days in culture (*n* = 8). **B.** Proliferation was not altered in cells harboring AKT^MYR^ or PTEN^shRNA^/AKT^MYR^. PTEN^shRNA^/AKT^MYR^/KRAS^G12V^ MOE cells had a reduction in proliferation compared to scrambled shRNA control (*n* = 5). **C.** and **D.** Anchorage independent growth occurred in several engineered MOE cells as demonstrated by colony formation *in vitro*, specifically cells with PTEN^shRNA^ in combination with other genetic alterations (denoted with arrows). Parental MOE cell (WT), SCR, and MOE cells harboring single genetic alterations did not form colonies. *p ≤ 0.05, **p ≤ 0.01 and ***p ≤ 0.001 as determined by *t*-test.

Stably modified MOE cells were characterized for phenotypic changes *in vitro*, specifically enhanced proliferation or anchorage independent growth. SRB assay determined PTEN^shRNA^ MOE had enhanced cell growth at 5 days (9.2±0.4 fold, *p* = 0.01) when compared with wildtype parental MOE cells (6.2±0.3 fold) or scrambled shRNA control MOE cells (7.1±0.5 fold, Figure 1A). MOE cells harboring PTEN^shRNA^ in combination with either p53^R273H^ or KRAS^G12V^ also displayed enhanced proliferation (10.6±1.1 fold, *p* = 0.02 and 10.8±0.7 fold, *p* = 0.01 respectively). The expression of p53^R273H^,KRAS^G12V^, or AKT^MYR^ alone in MOE cells did not alter proliferation (Figure 1B) [14]. Addition of AKT^MYR^ to generate PTEN^shRNA^/AKT^MYR^ MOE abrogated the enhanced growth seen with PTEN^shRNA^ alone. Further, MOE cells harboring triple manipulation; PTEN^shRNA^/AKT^MYR^/KRAS^G12V^ exhibited reduced cell growth (4.9±0.7 fold increase, *p* = 0.02).

*In vitro* transformative potential was investigated using a soft agar assay for anchorage independent growth. Wildtype and scrambled shRNA control MOE cells did not form colonies in soft agar (Figure 1C). Similarly, MOE cells with single genetic alterations were unable to form colonies [14]. However, MOE cells with PTEN knockdown in combination with another alteration did form colonies in soft agar (Figure 1D); PTEN^shRNA^/p53^R273H^ (5-fold increase compared to scrambled shRNA control), PTEN^shRNA^/AKT^MYR^ (3-fold increase), and PTEN^shRNA^/KRAS^G12V^ (22-fold increase). MOE cells stably transfected with three genetic alterations; PTEN^shRNA^/AKT^MYR^/KRAS^G12V^, were also transformed *in vitro*, with a 7-fold increase in colony formation.

### Transformed PTEN^shRNA^ MOE cell grafts mimic HGSC tumors in patients

**Figure 2:**
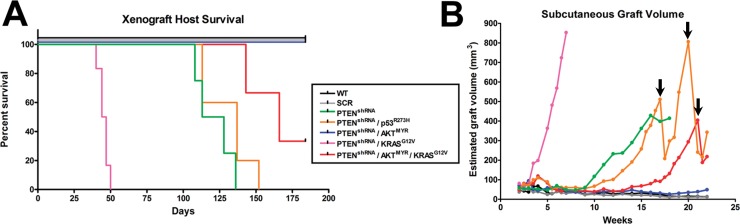
MOE cells form subcutaneous (s.c) and intraperitoneal tumors in an allograft model **A.** Several pathway modified MOE models were capable of tumorigenesis when grafted into athymic mice and had markedly reduced survival after 6 months *in vivo* (*n* = 4-6 mice per group). **B.** S.C. tumor growth was tracked via weekly caliper measurements. No growth was seen in parental (WT) or scrambled shRNA (SCR) control cell lines whereas pathway modified MOE cells formed large s.c. tumors. Arrows denote where animals were humanely sacrificed due to tumor burden, which modified the average tumor size.

To test tumorigenic potential of MOE cells *in vivo*, genetically engineered MOE cell lines and parental wildtype and scrambled shRNA controls were allografted into athymic mice subcutaneously (s.c) and intraperitoneally (i.p.) and tumorigenesis was assessed over a 6 month period (Figure 2A). Mice grafted with wildtype or scrambled shRNA control MOE had no evidence of tumor burden. MOE with single genetic changes to either p53^R273H^ or KRAS^G12V^ also did not form disease in athymic mice [14]. However, several MOE models generated tumors. Subcutaneous tumor growth was tracked via weekly caliper measurement (Figure 2B). When tumor burden reached a moribund stage animals were sacrificed, s.c. tumors weighed and i.p. disease assessed (Table 1).

**Table 1:** Summary of MOE allografted animal survival and tumor formation

MOE Cell Type	Survival (days)	S.C. Tumor Weight (mg)	Peritoneal Disease
Wildtype (WT)	no mortality	N/A	N/A
Scrambled shRNA (SCR)	no mortality	N/A	N/A
PTEN^shRNA^	147 ± 21	376.8 ± 107.1	100% (4/4)
PTEN^shRNA^ / p53^R273H^	130 ± 8	771.1 ± 198.1	60% (3/5)
PTEN^shRNA^ / AKT^MYR^	no mortality	69.93 ± 14.1	40% (2/5)
PTEN^shRNA^ / KRAS^G12V^	45 ± 1	520.1 ± 38.9	100% (6/6)
PTEN^shRNA^ / AKT^MYR^ / KRAS^G12V^	164 ± 8	347.5 ± 88.7	67% (4/6)

**Figure 3:**
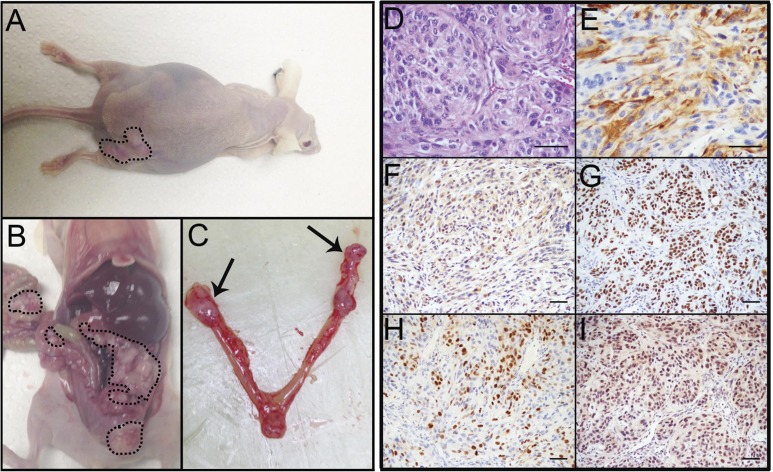
PTENshRNA MOE form high-grade carcinoma *in vivo* **A.** PTEN^shRNA^ MOE grafted mice formed large s.c. tumors (dashed outline) and **B.** disseminated i.p. disease (dashed outline). **C.** Notably, MOE tumors spread to the ovaries of grafted animals (arrows). Immunohistochemical analysis of PTEN^shRNA^ MOE via **D.** H&E, **E.** CK8, **F.** PAX8, **G.** WT1, **H.** p53, and **I.** Ki67 staining determined the tumors to be moderately differentiated high-grade carcinomas of oviductal epithelial origin. Scale bars equal 50 microns.

PTEN^shRNA^ MOE cells formed large s.c. tumors (376.8±107.1 mg, Figure 3A) and were sacrificed at 147±21 days post grafting. Further, PTEN^shRNA^ MOE formed tumor explants throughout the peritoneal cavity (4/4 animals, 100%, Figure 3B), including spread to the reproductive tract (4/4 animals), G.I./omentum (4/4 animals), and the diaphragm (3/4 animals, Table 2) Further, kidney, liver and lung were relatively disease free, a disease pattern that is common in patients, demonstrating that malignancy of oviductal epithelial origin mimics HGSC peritoneal colonization. In addition to the analogous disease spread, one of the four PTEN^shRNA^ MOE host animals (25%) also developed ascites fluid, another hallmark of HGSC tumorigenesis in women. When host reproductive tracts were excised, metastasis into the bursa and spread to the ovaries was noted (Figure 3C).

**Table 2:** Immunohistochemical analysis on MOE-derived tumors

	PTEN^shRNA^	PTEN^shRNA^ / p53^R273H^	PTEN^shRNA^ / KRAS^G12V^	PTEN^shRNA^ / AKT^MYR^	PTEN^shRNA^ / AKT^MYR^/KRAS^G12V^
CK8	+	+	+	-	+
PAX8	+	+	+	+	+
WT1	+	+/−	+	-	+/−
Ki67	50-60%	40-50%	60-70%	20-30%	30-40%
p53	1+ / 2+	1+	1+	-	-

Immunohistochemical analysis of PTEN^shRNA^ MOE derived tumors and subsequent pathological analysis confirmed the tumors were moderately differentiated high-grade carcinoma of oviductal epithelial origin. This was determined by moderate nuclear atypia seen in H&E staining (Figure 3D). Tumors consisted of epithelioid cells, primarily solid and nesting growth patterns with areas of poorly formed glandular structure. Cytokeratin 8 (CK8) and PAX8 staining (Figure 3E-3F) confirmed the tumors to be of oviductal epithelial origin. PTEN^shRNA^ MOE derived tumors also expressed the serous marker WT1 (Figure 3G), were highly proliferative (50-60% Ki-67 positive, Figure 3H) and demonstrated stabilized p53 protein expression via strong and diffuse immunoreactivity (Figure 3I).

### Mutation in p53 does not enhance PTEN^shRNA^ MOE phenotype

**Figure 4:**
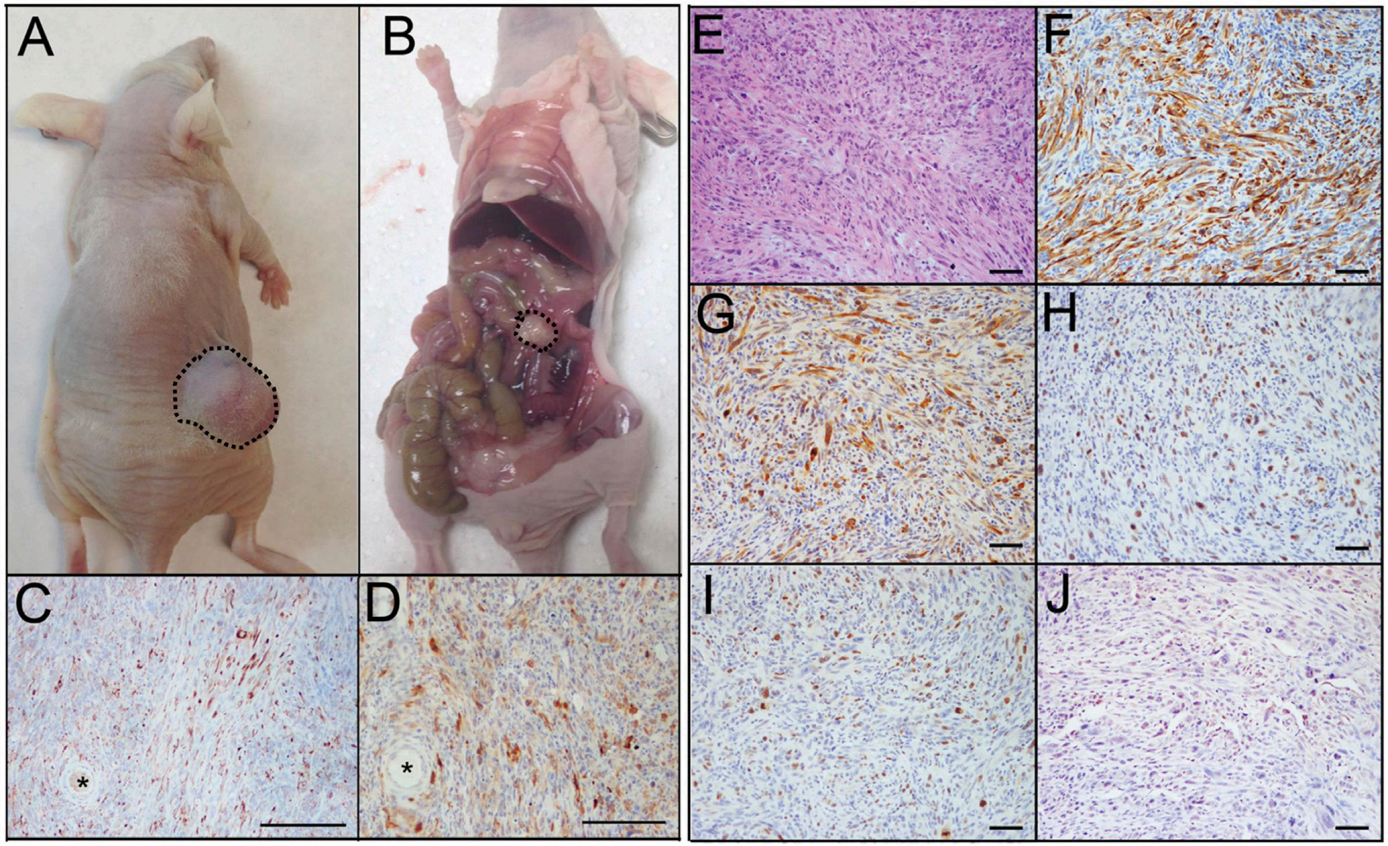
PTENshRNA/p53R273H MOE form high-grade carcinomas with reduced i.p. spread compared to PTENshRNA MOE **A.** As with PTEN^shRNA^-alone MOE, PTEN^shRNA^/p53^R273H^ MOE grafted mice formed large s.c. tumors (dashed outline) but **B.** had reduced i.p. disease (dashed outline). **C.** Despite the reduction in i.p. tumor explants, PTEN^shRNA^/p53^R273H^ MOE, staining for CK8 and **D.** PAX8 preferentially formed disease on the ovaries, with tumor invasion engulfing oocytes (*). Immunohistochemical analysis of PTEN^shRNA^/p53^R273H^ MOE via **E.** H&E, **F.** CK8, **G.** PAX8, **H.** WT1, **I.** Ki67, and **J.** p53 staining determined the tumors to be poorly differentiated high-grade sarcomatoid carcinoma. Scale bars equal 200 microns (C-D) and 50 microns (E-J).

Animals grafted with PTEN^shRNA^/p53^R273H^ MOE cells had similar s.c. disease formation (Figure 4A) and survival rates to that of PTEN^shRNA^ MOE grafted animals (130±8 days post grafting). However, peritoneal tumor burden was reduced, as not all PTEN^shRNA^/p53^R273H^ allograft mice presented with peritoneal disease (3 of 5 animals (60%), Figure 4B). Of the mice that developed i.p. disease, fewer malignant explants were found. Despite fewer sites of dissemination, animals with peritoneal disease had tumors in their ovaries (Figure 4C-4D), with infiltration noted via CK8 and PAX8 positive tumor cells engulfing the ovaries and leaving follicular structures surrounded by MOE-derived tumors.

PTEN^shRNA^/p53^R273H^ MOE derived tumors were pathologically assessed and determined to be poorly differentiated, high-grade tumors with features of sarcomatoid carcinoma. Tumor cells had high nuclear atypia with little to no glandular structure (Figure 4E). Cells were of MOE origin, characterized by their diffuse immunoreactivity for CK8 and PAX8 (Figure 4F-4G), serous marker WT1 expression was variable (Figure 4H). PTEN^shRNA^/p53^R273H^ tumors were highly proliferative (40-50% of cells staining positively for Ki67, Figure 4I) and expressed stabilized p53 (Figure 4J).

### Activation of KRAS enhances PTEN^shRNA^ phenotype

**Figure 5:**
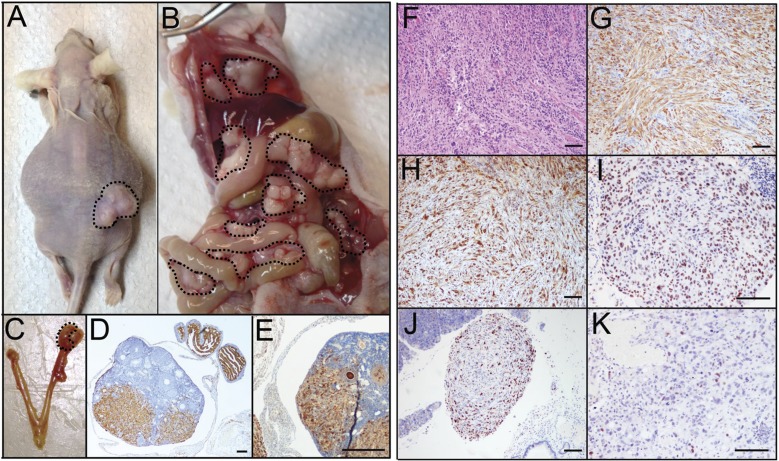
PTENshRNA/KRASG12V MOE give rise to highly-aggressive carcinomas with advanced i.p. spread **A.** PTEN^shRNA^/KRAS^G12V^ MOE grafted mice quickly developed large s.c. tumors (dashed outline) and some hosts developed ascites fluid. **B.** i.p. explants were dispersed throughout the peritoneal cavity including the G.I. tract and diaphragm (dashed outline). **C.** As with PTEN^shRNA^ and PTEN^shrNA^/p53^R273H^ grafted animals ovarian tumors were noted staining positively for **D.** CK8 and **E.** PAX8. Immunohistochemical analysis of PTEN^shRNA^/KRAS^G12V^ MOE via **F.** H&E, **G.** CK8, **H.** PAX8, **I.** WT1, **J.** p53, and **K.** Ki67 staining determined the tumors to be undifferentiated high-grade carcinomas of oviductal epithelial origin. Scale bars equal 200 microns (D-E) and 50 microns (F-K).

The addition of a mutation in KRAS (G12V) to PTEN^shRNA^ (PTEN^shRNA^/KRAS^G12V^) enhanced MOE cell carcinogenesis, with an average survival of 45±1 days. Large s.c. tumors developed quickly (520.1±38.9 mg, Figure 5A). Ascites fluid (1/6 animals (17%)), vast peritoneal disease spread (6/6 animals, 100%, Figure 5B), and reproductive explants (6/6 animals, 100%, Figure 5C) were found in PTEN^shRNA^/KRAS^G12V^ grafted hosts. Tumor explants were noted in the ovaries in the majority of animals with i.p. disease, while oviducts remained disease free in MOE grafted mice (Suppl. Fig. 2).

Immunohistochemical analysis of PTEN^shRNA^/KRAS^G12V^ MOE derived tumors determined them to be high-grade carcinomas of oviductal epithelial origin. Tumors had solid and sheath growth patterns with high-grade and pleomorphic nuclear atypia, and brisk of mitosis (Figure 5F). As with previous MOE grafts, PTEN^shRNA^/KRAS^G12V^ MOE derived tumors had strong and diffuse immunoreactivity for CK8, PAX8, and WT1, consistent with oviductal secretory cell origin (Figure 5G-5I). PTEN^shRNA^/KRAS^G12V^ tumors were highly proliferative (60-70% Ki67 staining, Figure 5J), which likely contributed to enhanced aggression of the disease. p53 was enhanced but varied from case to case (Figure 5K).

### Myristolation of AKT diminishes MOE tumor phenotype

**Figure 6:**
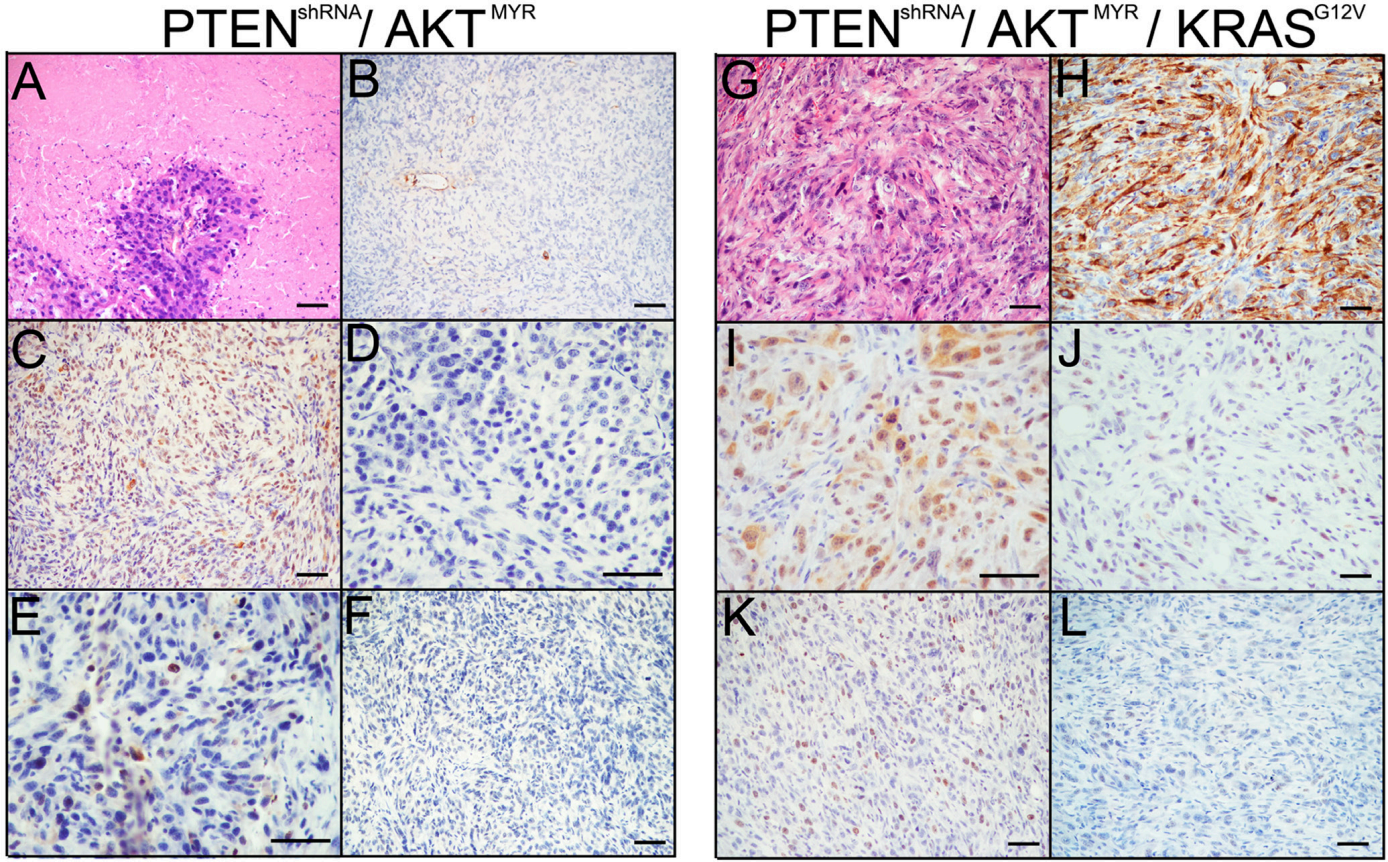
MOE with AKTMYR had reduced tumor phenotypes The addition of AKT^MYR^ to PTEN^shRNA^ in PTEN^shRNA^/AKT^MYR^ MOE grafted mice generated a less aggressive phenotype. Immunohistochemical analysis of PTEN^shRNA^/AKT^MYR^ MOE via **A.** H&E, **B.** CK8, **C.** PAX8, **D.** WT1, **E.** p53, and **F.** Ki67 staining determined the tumors to be full of necrotic and regressing cells as well as proliferating cells and reduction of epithelial markers, resulting in poorly differentiated high-grade mesenchymal-like carcinomas. Similarly, PTEN^shRNA^/AKT^MYR^/KRAS^G12V^ MOE had a reduced phenotype compared to either PTEN^shRNA^ or PTEN^shRNA^/KRAS^G12V^ MOE cells. Pathological evaluation of tumors stained for via **G.** H&E, **H.** CK8, **I.** PAX8, **J.** WT1, **K.** p53, and **L.** Ki67 revealed the PTEN^shRNA^/AKT^MYR^/KRAS^G12V^ derived tumors were poorly differentiated high-grade epithelioid sarcomas. Scale bars equal 50 microns.

Interestingly, the addition of AKT^MYR^ to PTEN^shRNA^ (PTEN^shRNA^/AKT^MYR^) or in combination with PTEN^shRNA^ and KRAS^G12V^ (PTEN^shRNA^/AKT^MYR^/KRAS^G12V^) resulted in reduced tumor burden. PTEN^shRNA^/AKT^MYR^ grafts were not lethal after six months, although s.c. tumors 5-fold smaller than those found in PTEN^shRNA^ MOE mice, were recovered at the time of sacrifice (69.93±14.1 mg). Two of five animals (40%) had singular metastatic explants, a drastic reduction in tumor spread compared to PTEN^shRNA^ grafted mice. Pathological assessment determined PTEN^shRNA^/AKT^MYR^ tumors were poorly differentiated high-grade sarcomatoid carcinoma. H&E staining revealed extensive tumor necrosis and apoptosis (Figure 6A). Tumors were hypercellular with high nuclear to cytoplasmic ratio, and features of ‘small-cell like’ morphology. Further, tumor cells had reduced CK8 and low PAX8 expression (Figure 6B-6C). The serous marker WT1 was also lost (Figure 6D). PTEN^shRNA^/AKT^MYR^ s.c. tumors displayed low mitotic indices (20-30% Ki67 staining, Figure 6E), and no stabilized p53 expression (Figure 6F).

PTEN^shRNA^/AKT^MYR^/KRAS^G12V^ grafts also produced a diminished tumor phenotype when compared to either PTEN^shRNA^ or PTEN^shRNA^/KRAS^G12V^ grafted animals, identified as poorly differentiated high-grade sarcomatoid carcinoma, with an over three-fold increase in survival compared with PTEN^shRNA/^ KRAS^G12V^ grafted mice (164±8 days). Only four of six (67%) PTEN^shRNA^/AKT^MYR^/KRAS^G12V^ grafted mice were moribund prior to the 6 month experimental timepoint. Resulting s.c. tumors were comparable to other lethal MOE allografts (347.5±88.7 mg). Four of six (67%) PTEN^shRNA^/AKT^MYR^/KRAS^G12V^ grafted mice formed peritoneal explants; however disease was reduced compared to PTEN^shRNA^ or PTEN^shRNA^/KRAS^G12V^, with no animal harboring more than two peritoneal explants. Both the reproductive tract and the pancreas remained preferential sites of tumor colonization (Table 3).

**Table 3:** Sites of MOE peritoneal explant colonization

	PTEN^shRNA^	PTEN^shRNA^/p53^R273H^	PTEN^shRNA^/KRAS^G12V^	PTEN^shRNA^/AKT^MYR^	PTEN^shRNA^/AKT^MYR^/ KRAS^G12V^	PTEN^shRNA/^ KRAS^G12V^ SYNGENEIC
Reproductive Tract	4 / 4	3 / 5	6 / 6	0 / 5	3 / 6	0 / 5
G.I. Tract / Omentum	4 / 4	3 / 5	6 / 6	1 / 5	1 / 6	3 / 5
Diaphragm	3 / 4	1 / 5	4 / 6	0 / 5	0 / 6	2 / 5
Pancreas	4 / 4	2 / 5	4 / 6	2 / 5	4 / 6	4 / 5
Kidney	0 / 4	0 / 5	1 / 6	0 / 5	0 / 6	0 / 5
Liver/Lung	1 / 4	0 / 5	1 / 6	0 / 5	0 / 6	1 / 5
Ascites	1 / 4	0 / 5	1 / 6	0 / 5	0 / 6	5 / 5

As with PTEN^shRNA^/AKT^MYR^, tumors were high-grade carcinoma with notable nuclear atypia (Figure 6G). PTEN^shRNA^/AKT^MYR^/KRAS^G12V^ tumors were immunoreactive for CK8 and PAX8 although expression was diminished compared to lethal MOE models (Figure 6H-6I). WT1 expression and proliferation were reduced (30-40% proliferation, Figure 6J-6K). Moreover, p53 expression was not stabilized (Figure 6L). These data demonstrate that although enhanced phosphorylation of AKT is seen in HGSC tumors, enhanced AKT signaling does not phenocopy loss of PTEN, with notable alterations in tumor spread, p53 stabilization, and expression of serous markers such as WT1.

### Therapeutic targeting and syngeneic allografts of PTEN^shRNA^/KRAS^G12V^ MOE cells

**Figure 7:**
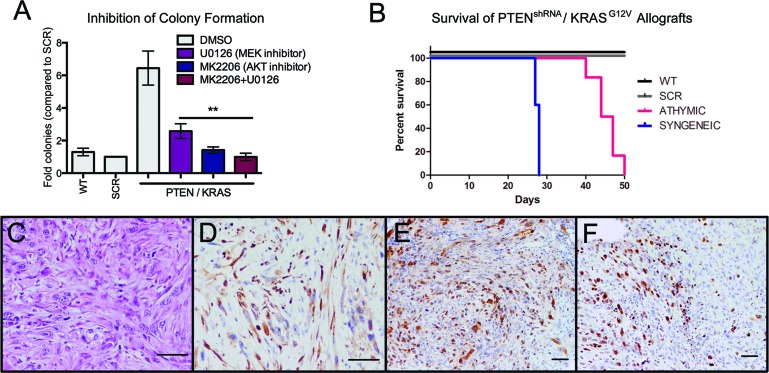
MOE cells as preclinical models for therapeutic development **A.** Treatment with the AKT inhibitor (MK2006 (10 μM), counteracts PTEN suppression) or with the MEK inhibitor (U0126 (10 μM), inhibits signaling downstream of KRAS) represses colony formation in soft agar compared to vehicle-treated (DMSO) control (*n* = 6). **B.** Syngeneic PTEN^shRNA^/KRAS^G12V^ allografts have drastically reduced survival time compared to athymic PTEN^shRNA^/KRAS^G!2V^ allografts. **C.** Tumors from syngeneic PTEN^shRNA^/KRAS^G!2V^ allografted mice were similar in morphology to those of athymic hosts as determined via H&E staining and expression of **D.** CK8, **E.** PAX8, and **F.** Ki67 staining. Scale bars equal 50 microns.

PTEN^shRNA^/KRAS^G12V^ MOE cells could be utilized to test novel targeted therapeutics prior to human clinical trials. To highlight potential pathways for therapeutic intervention, PTEN^shRNA^/KRAS^G12V^ MOE cells were treated with specific chemical inhibitors downstream of genetic alterations and subjected to soft agar assay. MK2206 (10μM), an allosteric AKT inhibitor, was utilized to diminish the effects of PTEN silencing, and U0126 (10μM), a MEK1/2 inhibitor, was utilized to inhibit uncontrolled signaling downstream of KRAS mutation (Figure 7A). U0126 reduced colony formation by 60%, MK2206 by 78% and combined inhibitor treatment by 84%, demonstrating both pathways are required for colony formation, inhibition of which is able to suppress *in vitro* transformation.

Additionally, PTEN^shRNA^/KRAS^G12V^ cells were utilized to demonstrate the potential for MOE cells as a syngeneic model, allowing for preclinical investigation of novel therapeutics in the presence of an immune system. FVB mice were i.p. allografted with PTEN^shRNA^/KRAS^G12V^ MOE and developed high-grade carcinomas. Interestingly, immno-competent FVB host animals had reduced survival (28±0.2 vs 45±1 days, Figure 7B). This was likely due to increased occurrence of ascites fluid build-up, seen in all syngeneic grafted animals. Tumor explants were of a similar morphology and disease pattern to that seen previously in athymic allografted animals (Figure 7C-7F, Table 3). MOE models could be utilized for therapeutic development in the presence of an immune-competent *in vivo* environment.

## DISCUSSION

To address origin and initiation factors leading to HGSC tumorigenesis, murine oviductal epithelial cells (MOE) were modified to stably express alterations in key pathways affected in HGCS patients. This study demonstrated that oviductal epithelium can give rise to tumors similar to those identified in HGSC patients from characterized genetic drivers of disease. *In vitro* characterization of MOE cells demonstrated specific genetic alterations, namely PTEN^shRNA^ alone and in combination with KRAS^G12V^, p53^R273H^, and AKT^MYR^ induced phenotypic changes in proliferation and anchorage independent growth. *In vivo* characterization in allografted athymic and subsequent syngeneic murine model produced MOE derived high-grade carcinomas, which resembled HGSC found in women both in pathological staining and pattern of disease spread. These data support the fallopian tube/oviduct as a potential origin for HGSC, expand upon previous studies of key genetic regulators in human and murine models, and provide a novel immune-competent model for biomarker and therapeutic development.

Silencing PTEN alone initiated tumor formation from MOE cells. Studies using PAX8 to drive Cre-recombinase mediated deletion of PTEN did so only in combination with other genes such as BRCA1/2 or mutant p53 R270H [4]. Importantly, recent evidence indicates that PTEN loss is more common than initially thought in HGSC when epithelium are isolated away from stromal contaminants [15]. This is the first publication to demonstrate that PTEN silencing alone in a murine model of oviductal epithelium can generate tumors. PTEN loss also drives tumor formation from the endometrium, thyroid, and prostate epithelium indicating that multiple types of hormone responsive epithelium are dependent on expression of PTEN to block tumor formation [16]. Another Cre-recombinase murine model driven by MISRII to delete PTEN alone did not demonstrate a phenotype, but this was likely due to Cre-mediated excision in the oviductal stroma and ovarian surface epithelium rather than the oviductal epithelium and suggests that PTEN governs different pathways in these cell types [6, 17]. In agreement with this hypothesis, findings from our lab indicated that PTEN silencing in murine OSE was unable to generate soft agar colonies [18]. Moreover, a publication recently attempted to intraoviductally inject adenovirus into MUC1/KRAS/PTEN floxed mice, which resulted in high-grade endometrioid carcinoma; however, intraoviductal injections can be leaky, often infecting the ovarian surface, and as previous reports suggest, oviductal infectivity is limited *in vivo* and in 3D *in vitro* cultures [5, 7].

The incorporation of mutant p53 into PTEN^shRNA^ cells did not enhance proliferation or tumor forming potential observed with PTEN^shRNA^ alone. These data are consistent with studies performed in human samples that indicate that mutation in p53 does not correlate with changes in tumor progression or survival [19]. In fact, mutation of p53 reduced the extent of tumor spread in mice with PTEN^shRNA^ suggesting that p53 mutation functions in a very context specific manner. These results are similar to the PAX8-Cre models where the addition of mutant p53 to PTEN and BRCA did not dramatically impact survival [4]. Previous reports suggest that mutation of p53 can slow migration as compared to wildtype [20] and that wildtype p53 in OSE derived tumors enhances survival [21]. However, mutation in p53 enhances tumor formation in the APC/PTEN model of OSE derived endometrioid cancer providing evidence that both the mutation landscape and the cell of origin impact the functional context of p53 mutation and resulting tumor histotype [22]. The current study focused on one DNA mutation of p53, R273H, the most common mutation in HGSC. However, many other mutations are seen in HGSC, making it difficult to decipher a general role for p53 and how it impacts tumorigenesis from the oviduct.

Data from this study highlight the importance of context in the generation of disease; namely the cell of origin, the genetic landscape/specific mutations, and the site of tumor development, especially when allografted. This is highlighted by PTEN silencing alone in the OSE, which does not drive tumor formation [23], whereas in the oviductal epithelium PTEN loss is sufficient for tumorigenesis. We speculate, this variation in susceptibility may be due in part to disparity between the two cell types; oviductal epithelium, which may be more representative of a true epithelium and is highly similar to that of other PTEN susceptible epithelial populations, such as endometrial epithelium [15], as compared to the OSE, which is developmentally less differentiated and more characteristic of a mixed mesothelium [24]. The molecular signaling landscape also impacts tumor formation, as evidenced by PTEN^shRNA^ alone and in combination with KRAS mutation developing more aggressive peritoneal disease as compared to PTEN^shRNA^ in combination with p53 mutation or activation of AKT. Furthermore, tumor histology was influenced by the site of grafting as PTEN^shRNA^ tumors appeared more epithelioid when allografted into the peritoneal cavity, a more orthotopic model of HGSC as compared to the subcutaneous space. The cell of origin from which a tumor is derived further impacts the spectrum of spread. The ovary was a common site of infiltration of MOE models that formed peritoneal explants. In contrast, despite cell models being derived from the oviduct, colonization in the oviduct was not seen. In addition to the ovary, the gynecologic tract in general was a site of tumor adhesion and growth suggesting that the ovaries and uterus express receptors or growth factors that help to stimulate colonization. As the rodent ovary is surrounded by a bursa, identification of MOE derived ovarian tumors indicates metastasis through the bursa or through the lymphatic or hematopoietic systems to penetrate the ovarian cortex after peritoneal injection. Taken together these data highlight the context of disease, in particular the cells impacted, the specific pathways altered, and the site of tumor growth will influence the phenotype and therefore the future use of models for preclinical testing.

Overall these findings indicate that the secretory epithelium of the oviduct can form tumors that resemble the human disease through genetic manipulation of pathways that are frequently modified in HGSC. PTEN silencing alone was sufficient to drive widespread disease and was further enhanced with activation of KRAS but not mutation of p53. KRAS mutation enhanced PTEN^shRNA^ phenotype, while AKT^MYR^ blunted the peritoneal spread seen in PTEN^shRNA^ and PTEN^shRNA^/KRAS^G12V^ MOE cells. These data are intriguing as they suggest PTEN^shRNA^ does not phenocopy AKT^MYR^ and may have functionality independent of PI3K/AKT signaling. This is supported by previous studies which demonstrated PTEN loss results in reduction of p53 expression in tissues and cells [25]. Further, it is postulated p53 transcriptional activity is modulated by direct PTEN interaction with p300 and p53 in the nucleus, a mechanism independent of the AKT pathway [26]. However, the difference between PTEN^shRNA^ and AKT^MYR^ in the present study may also be the result of artificial activation of AKT via myristolation, which via lipid modification, forces AKT to be presented at the cell membrane and thus constitutively activated. As a result, it is possible actions of AKT away from the cell membrane, such as in the nucleus where it is postulated to interact with FOXO family transcription factors may be suppressed in this model, thereby creating the differences seen between PTEN suppression and AKT activation [27]. Further, the construct utilized by this study activates AKT1, and although it is thought that AKT2 and 3 isoforms can compensate, evidence suggests they play distinct roles in cancer progression [28, 29]. Thus it may be beneficial to utilize a cell line incorporating all AKT isoforms, such as PI3K activation, to further clarify possible phosphatase-independent mechanisms by which PTEN promotes tumorigenesis. Despite these differences, colony formation was disrupted with an AKT small molecule inhibitor in cells with absent PTEN. This was also demonstrated with the MEK inhibitor U0126 downstream of KRAS function. Recent reports demonstrate FOXO3 is differentially expressed in p53 signatures and STICs and correlated with changes in KRAS and PTEN signaling that may underlie alterations from a benign to a malignant phenotype [30].

The novel MOE cell model created in this study has demonstrated that context of carcinogenesis is extremely impactful on the resulting disease, in particular the cell of origin and genetic alterations leading to tumorigenesis. These models can be used to investigate secreted factors that might serve as biomarkers, to directly compare against similar OSE models [31] and can be allografted sygeneically, thereby allowing for the study of tumorigenesis in the presence of immune function and immune-modulating therapies. Clarifying the mechanisms contributing to HGSC formation will inform the development of targeted screening, prevention and therapy, thereby impacting HGSC patient prognosis and survival.

## MATERIALS AND METHODS

### Cell culture

MOE were isolated from the oviducts of cycling FVB/N mice in accordance with the guidelines of the Canadian Council on Animal Care and with institutional approval by the University of Ottawa. Cells were clonogenically grown from single cells and confirmed as oviductal epithelium by expression of PAX2, PAX8, and OVGP1 proteins. MOE were cultured as previously described [18].

### Stable cell development

Parental MOE cells were transfected via TransIT LT1 (Mirus Bio, Madison, WI) according to the manufacturer's instructions with plasmids containing targets of interest (Suppl. Table 1). Cells were clonogenically isolated via antibiotic selection (Suppl. Table 1) and validated via qRT-PCR and western blotting.

### Western blotting

Cell lysates (30μg) were run on 12% SDS-PAGE gels and transferred to nitrocellulose membranes (Fisher Scientific, Pittsburgh, PA). Membranes were incubated with antibodies targeting proteins of interest overnight at 4° C (Suppl. Table 2) prior to detection via horseradish peroxidase-conjugated secondary antibody (1:1000, Cell Signaling, Cambridge, MA) and SuperSignal West Femto substrate (Thermo Scientific, Rockford, IL, USA).

### Proliferation assay

Sulforhodamine B (SRB) assay was utilized to determine cell density as previously described [32]. Briefly, MOE were plated at 1,000 cells per well in a 96-well plate and fixed at varying time points as described. Plates were probed with SRB and cell density determined by comparison to a cell-matched control plate fixed 2 hours post plating.

### Soft agar colony formation assay

Anchorage independent growth was assessed via soft agar assay as previously described [18]. MOE cells were seeded at a density of 1.5×10^5^ cells per well in 0.35% agarose (Sigma Aldrich, St. Louis, MO) / DMEM (Life Technologies, Carlsbad, CA) in a 24-well plate. Cells were incubated for 2 weeks, with media changed every 4 days (DMEM, 1x penicillin/streptomycin (Gibco, Carlsbad, CA), 4% FBS (Life Technologies)). Data was blinded prior to quantification via ImageJ software (NIH, Bethesda, MD). Subsequent assays utilizing inhibitors (MK2206 (10μM) and U0126 (10μM)) with DMSO as vehicle control, treatments were refreshed every 4 days with overlying media.

### Study approval

All animals were treated in accordance with the NIH guidelines for Laboratory Animals and established Institutional Animal Use and Care protocol at the University of Illinois at Chicago.

### Allografting

Athymic mice were acquired from (Taconic, Germantown, NY) and allografted with MOE cells including the parental/wildtype (WT) and scrambled shRNA (SCR) control lines 2×10^6^ cells in 300μL PBS:matrigel (1:1 vol/vol) subcutaneously (s.c) and intraperitoneally (i.p.) 1×10^7^ cells in 300μL PBS. Animal weight and s.c. tumor growth (via caliper measurement) were tracked weekly and animals sacrificed when tumor burden or general health was determined to be moribund. Syngeneic grafts were performed in FVB/N mice via i.p. injection of 1×10^7^ cells in 300μL PBS. Subcutaneous grafts were not performed in immune competent hosts.

### Tissue collection and analysis

At the time of sacrifice, s.c. tumors were dissected and weighed, evidence of i.p. disease noted and collected along with reproductive and gastrointestinal tracts for further investigation. Tissues were fixed in 4% paraformaldehyde before dehydration in ethanol and xylene prior to paraffin embedding. Immunohistochemistry was performed as previously described [33]. Briefly, tissues were sectioned and rehydrated in a gradient of ethanol prior to antigen retrieval and peroxidase block. Sections were incubated in primary antibody overnight at 4° C (Suppl. Table 2) before detection via biotinylated secondary antibody (1:200, Vector Laboratories (Burlingame, CA) and ABC peroxidase (Vector Laboratories). Targets were visualized via 3,3′-diaminobenzidine (DAB, Vector Laboratories) and counterstained with hematoxylin.

### Statistics

All data are displayed as mean ± standard error. Significance for *in vitro* proliferation and anchorage independent growth assays was determined via paired t-test utilizing Prism software (GraphPad, La Jolla, CA). All data sets were analyzed for significant outliers by Grubbs' test of deviation.

## SUPPLEMENTARY FIGURES AND TABLES


