# Supplementary material for: Tumorigenesis and peritoneal colonization from fallopian tube epithelium
Source: Oncotarget. 2015 May 4;6(24):20500–12. doi: 10.18632/oncotarget.3985 (PMC4653021; doi:10.18632/oncotarget.3985)
Supplement: Supplementary file 1 [file oncotarget-06-20500-s001.pdf]

Tumorigenesis and peritoneal colonization from fallopian tube epithelium

Supplementary Material

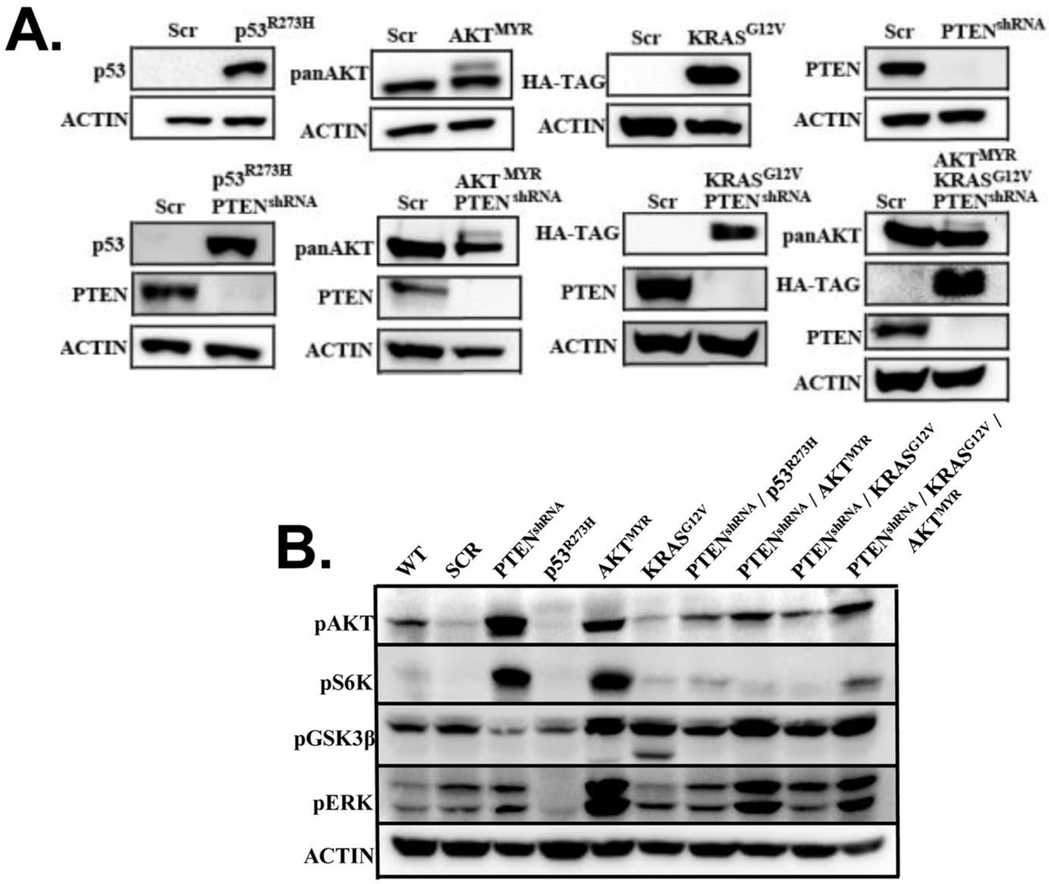

Supplementary Figure 1: Validation of genetically engineered MOE. A) Stable MOE cell lines were validated via western blotting to express targets of interest as compared to scrambled shRNA (SCR) transfected control MOE cells. Specifically, stabilized p53 expression, which is indicative of mutation, double-banding of pan-AKT which demonstrates myristolation, HA-Tag expression by the KRAS plasmid, and absence of PTEN expression when transfected with shRNA. **B)** Further, cell lines were evaluated for downstream signaling changes via western blotting, to demonstrate that both select targets and their signaling pathways were functionally altered. WT= wildtype parental MOE cells.

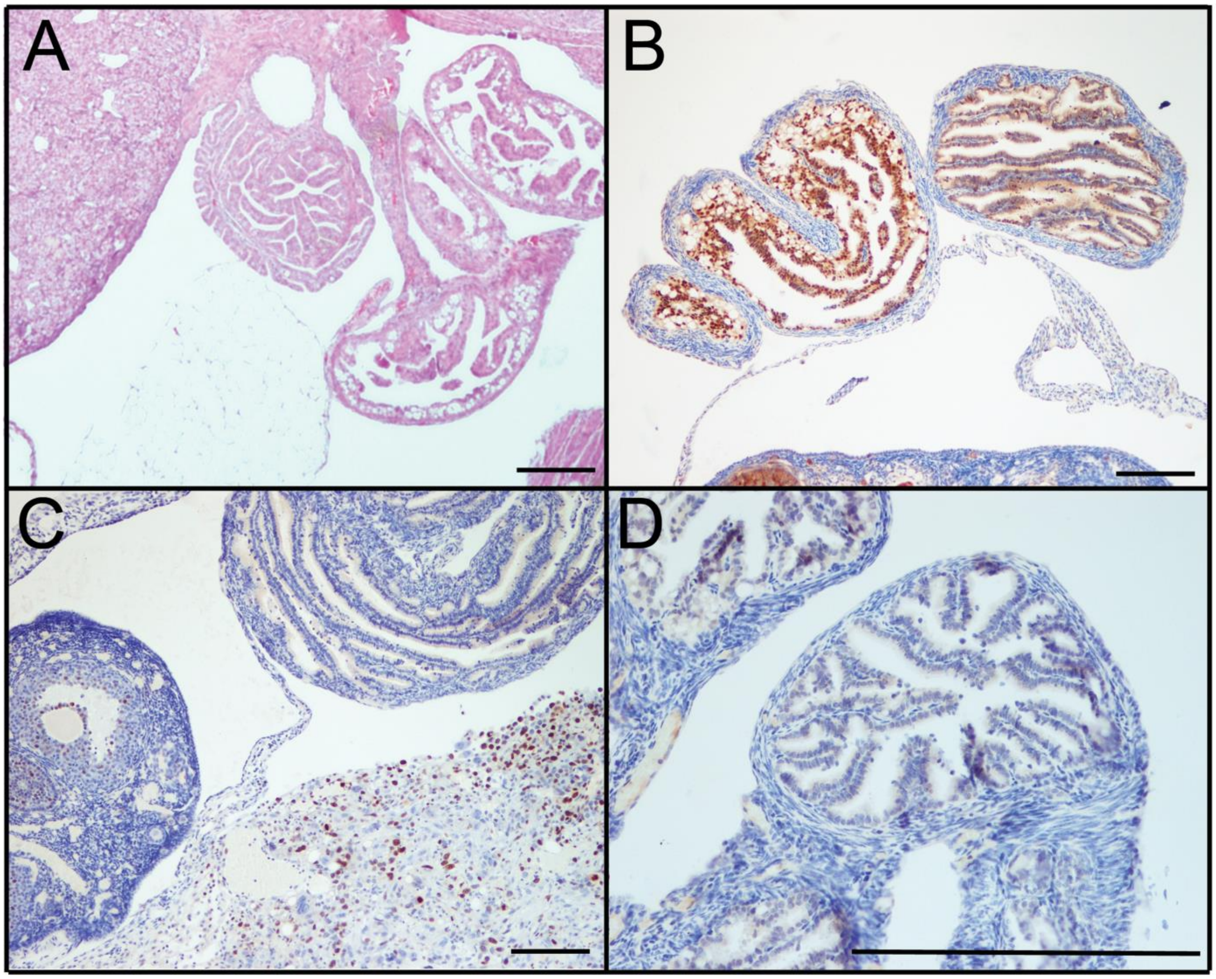

Supplementary Figure 2: Tumorigenic MOE do not metastasize to the oviduct. A) H&E staining demonstrated that although tumors were found on adjacent ovaries, no oviductal involvement was found in any MOE grafted mice. **B)** Oviducts remained normal, as shown by PAX8 staining, **C)** with no infiltration of highly proliferative (Ki67) tumors and **D)** no stabilization of p53. Scale bars equal 200 microns.

Supplementary Table 1: Plasmids utilized for MOE cell development

Target	Vector	Manufacturer	Antibiotic Maintenance
**Scrambled non-coding shRNA**	pLKO.1 puro	Sigma	0.5 µg/ml puromycin (Sigma)
**PTEN shRNA**	pLKO.1 puro	Sigma	0.5 µg/ml puromycin (Sigma)
**KRAS G12V**	pLenti-PGK-Hygro	Addgene (35633)	200 µg/ml hygromycin (Sigma)
**myristoylated AKT**	pcDNA3 Myr HAtag	Addgene (9008)	300 µg/ml neomycin (Sigma)
**p53 R273H**	pCMV-Neo-Bam	Addgene (16439)	300 µg/ml neomycin (Sigma)

Supplementary Table 2: Western blotting and immunohistochemical antibodies

Target	Manufacturer	Dilution Factor	Blocking Buffer
**Antibodies for western blotting:**			
**p53**	Santa Cruz	1:1000	5% milk-TBST
**pan AKT**	Cell Signaling	1:500	5% BSA-TBST
**HA-Tag**	Cell Signaling	1:500	5% milk-TBST
**PTEN**	Cell Signaling	1:1000	5% BSA-TBST
**phospho-AKT**	Cell Signaling	1:500	5% BSA-TBST
**phospho-S6K**	Cell Signaling	1:500	5% BSA-TBST
**phospho-GSK3β**	Cell Signaling	1:500	5% milk-TBST
**phospho-ERK**	Cell Signaling	1:1000	5% BSA-TBST
**ACTIN**	Sigma	1:1000	5% milk-TBST
**Antibodies for immunohistochemistry:**			
**CK8**	Iowa Hybridoma Bank	1:200	rabbit serum-TBS/BSA
**PAX8**	Proteintech	1:100	goat serum-TBS/BSA
**WT1**	Abcam	1:50	goat serum-TBS/BSA
**p53**	Santa Cruz	1:50	goat serum-TBS/BSA
**Ki-67**	Abcam	1:100	goat serum-TBS/BSA
